# Spt6-Spn1 interaction is required for RNA polymerase II association and precise nucleosome positioning along transcribed genes

**DOI:** 10.1016/j.jbc.2025.108436

**Published:** 2025-03-22

**Authors:** Boning Chen, Raghuvar Dronamraju, Whitney R. Smith-Kinnaman, Sarah A. Peck Justice, Austin J. Hepperla, Heather K. MacAlpine, Jeremy M. Simon, Amber L. Mosley, David M. MacAlpine, Brian D. Strahl

**Affiliations:** 1 Department of Pharmacology and Cancer Biology, Duke University Medical Center, Durham, North Carolina, USA; 2 Department of Biochemistry and Biophysics, University of North Carolina, Chapel Hill, North Carolina, USA; 3 Indiana University School of Medicine, Department of Biochemistry and Molecular Biology, Indianapolis, Indiana, USA; 4 Department of Biology, Marian University, Indianapolis, Indiana, USA; 5 Department of Genetics, University of North Carolina, Chapel Hill, North Carolina, USA; 6 Bioinformatics and Analytics Research Collaborative (BARC), University of North Carolina at Chapel Hill, Chapel Hill, North Carolina, USA; 7 Department of Data Science, Dana-Farber Cancer Institute, Boston, Massachusetts, USA; 8 Department of Biostatistics, Harvard T.H. Chan School of Public Health, Cambridge, Massachusetts, USA

**Keywords:** chromatin, histones, histone chaperone, nucleosomes, spn1, spt6, transcription, yeast

## Abstract

Spt6-Spn1 is an essential histone chaperone complex that associates with RNA Polymerase II (RNAPII) and reassembles nucleosomes during gene transcription. While the interaction between Spt6 and Spn1 is important for its histone deposition and transcription functions, a precise mechanistic understanding is still limited. Here, using temperature-sensitive alleles of *spt6* and *spn1* that disrupt their interaction in yeast, we show that the Spt6-Spn1 association is important for its stable interaction with the elongating RNAPII complex and nucleosomes. Using micrococcal nuclease (MNase)-based chromatin occupancy profiling, we further find that Spt6-Spn1 interaction is required to maintain a preferred nucleosome positioning at actively transcribed genes; in the absence of Spt6-Spn1 interaction, we observe a return to replication-dependent phasing. In addition to positioning defects, Spt6-Spn1 disrupting mutants also resulted in an overall shift of nucleosomes toward the 5′ end of genes that were correlated with decreased RNAPII levels. As loss of Spt6-Spn1 association results in cryptic transcription at a subset of genes, we examined these genes for their nucleosome profiles. These findings revealed that the chromatin organization at these loci is similar to other active genes, thus underscoring the critical role of DNA sequence in mediating cryptic transcription when nucleosome positioning is altered. Taken together, these findings reveal that Spt6-Spn1 interaction is key to its association with elongating RNAPII and to its ability to precisely organize nucleosomes across transcription units.

Histone chaperones are fundamental to genome organization and function (1, 2, 3). The evolutionarily conserved histone chaperone Spt6 (SUPT6H in humans) and its binding partner, Spn1 (IWS1 in humans), are essential chaperones that function in gene transcription (4). Studies from yeast to humans demonstrate that Spt6 associates with the phosphorylated and elongating form of RNA Polymerase II (RNAPII) along with other transcription elongation machinery, including the DRB Sensitivity-Inducing Factor (DSIF; Spt4-Spt5), the polymerase-associated factor 1 complex (Paf1C), Spt2, Elf1, and TFIIS (4, 5, 6, 7). In addition to promoting transcription elongation, Spt6 also functions with FACT to reassemble nucleosomes in the wake of RNAPII transcription (4, 5, 6). This cycle of nucleosome disruption and reassembly is not only vital for the ability of RNAPII to transcribe through chromatin but to also maintain chromatin integrity which is critical for genome maintenance.

Spt6, through its multiple domains, makes multiple contacts with RNAPII in addition to several of the aforementioned elongation factors. The interactions made by Spt6 are required for the stimulation of RNAPII elongation through chromatin (4). Recent Cryo-EM structures of RNAPII complexes containing Spt6 (and Spn1) have illuminated the many interactions that take place between these elongation factors in the elongating RNAPII complex and align with previous genetic analyses of the transcription complex (8, 9, 10). Spt6 and Spn1, along with DSIF, Spt2, Elf1, and TFIIS, cover almost the entire surface of RNAPII. Previous studies examining depletion and/or mutations of Spt6 revealed significant defects in RNAPII elongation, RNA processing, termination, and nucleosome organization and spacing in yeast, as well as increased cryptic transcription (4). Although Spt6 has been well-studied, there remain many unknowns in how it contributes to nucleosome reassembly and the promotion of transcription. Even less understood is the binding partner of Spt6, Spn1, which, like Spt6, contributes to transcription elongation and nucleosome reassembly (11, 12, 13, 14).

While Spt6 and Spn1 both associate with histones and share roles in nucleosome reassembly, genetic studies have determined that Spt6 and Spn1 also have distinct functions in transcriptional regulation (15, 16). Despite this, it is also clear that some functions of Spt6, such as nucleosome reassembly, are regulated by Spn1-Spt6 association—an interaction that is enhanced by the phosphorylation of the N-terminus of Spt6 by casein kinase II (CKII) (13, 17, 18, 19, 20). Here, we sought to provide further insights into the functional role of the Spt6-Spn1 interaction. We used conditional mutants of *spt6* and *spn1* (Fig. 1*B*) and ref (19) that target their interaction interface and are known to lead to their dissociation at non-permissive temperatures, causing defects in both chromatin organization and gene transcription. Using these mutants, we performed a rigorous mass spectrometry-based interaction analysis that revealed the importance of Spt6-Spn1 interaction for their ability to associate with elongating RNAPII and histones. On the genomic level, using MNase chromatin profiling, we show that disruption of Spt6-Spn1 interaction results in genome-wide nucleosome rotational realignment and/or shifting of ∼10.5 base pairs across actively transcribed genes. Further analysis found that Spt6-Spn1 disruption also resulted in a genome-wide shift of nucleosome density toward the 5′ ends of genes that were correlated with reduced RNAPII distribution. While mutants of *spt6* that prevent Spt6-Spn1 interaction are known to cause 5′ antisense transcription at a subset of genes (13, 17), our nucleosome profile mapping did not reveal a different nucleosome outcome at these 5′ ends that would account for the occurrence of antisense transcription, thus supporting sequence-specific events as being causal once an altered nucleosome pattern emerges. Taken together, these data suggest that an important function of Spt6-Spn1 association with RNAPII is to ensure proper transcription elongation and to precisely space and align/orient nucleosomes in the wake of RNAPII transcription.

**Figure 1:**
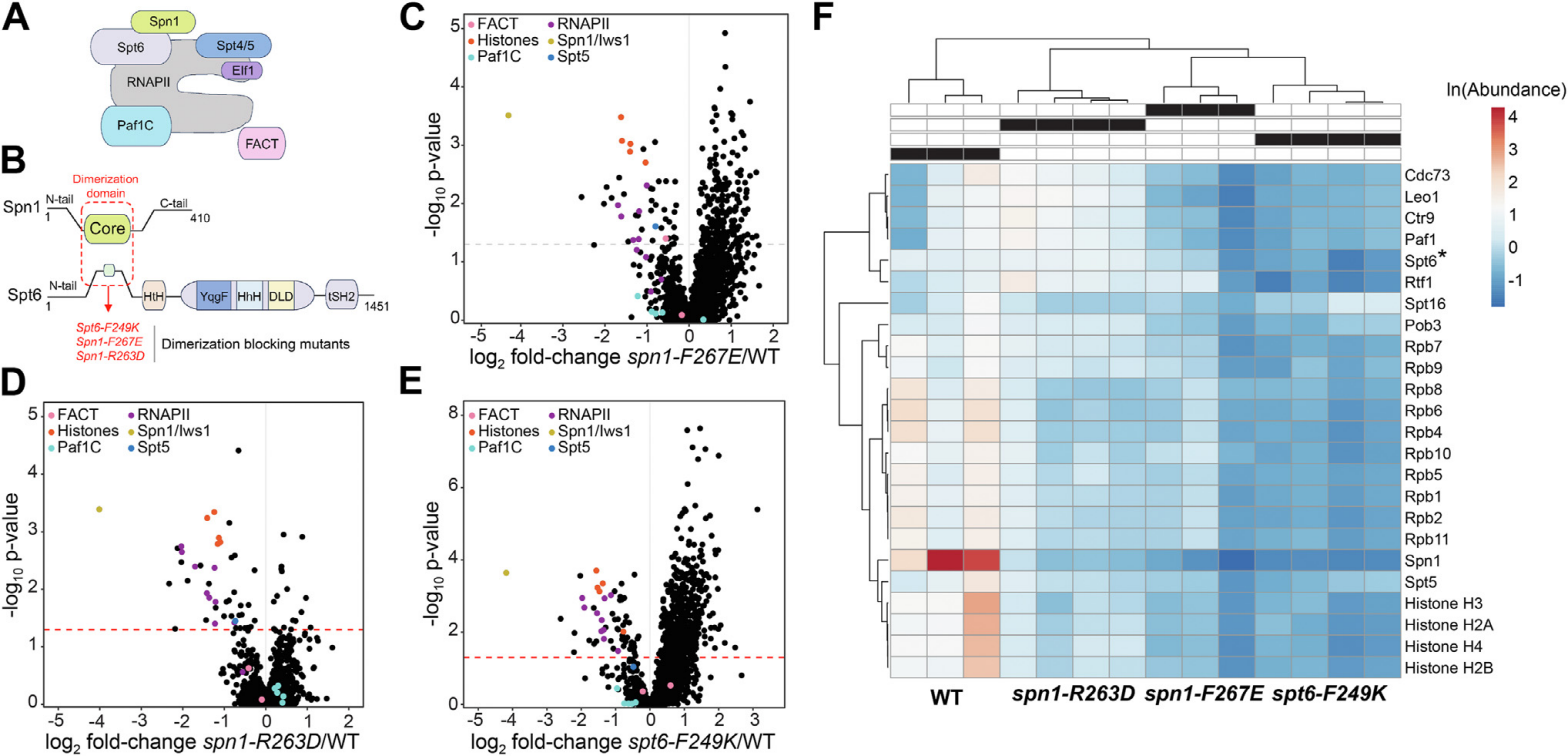
**Spt6-Spn1 interaction is required for histone and RNAPII association.***A*, schematic representation of Spt6-Spn1 association with RNAPII. *B*, domain structures of Spt6 and Spn1. *Red* outline highlights the Spt6-Spn1 dimerization domains and mutants previously shown to disrupt Spt6-Spn1 interaction that were used in this study. *C*–*E*, volcano plots of fold-change of Spt6-3XFLAG interactors relative to WT in (*C*) *spn1-F267E*, (*D*) *spn1-R263D*, and (*E*) *spt6-F249K*. X-axis is log_2_ fold-change of mutant/WT; y-axis is -log_10_*p*-value. 1963 data points are shown on each graph. Proteins of interest are highlighted and colored by complex, as shown in the key. Significance threshold was set at *p*-value < 0.05 and is indicated by a *dotted line*. A total of 259 proteins had significantly changed interactions with Spt6 in *spn1-F267E* (*C*); 62 proteins had significantly changed interactions with Spt6 in *spn1-R263D* (*D*); and 567 proteins had significantly changed interactions with Spt6 in *spt6-F249K* (*E*). *F*, heatmap and clustering analysis (ClustVis, ref (47)) of protein abundance in Spt6-3XFLAG WT (n = 3), *spn1-F267E* (n = 3), *spn1-R263D* (n = 4), and *spt6-F249K* (n = 4). Shown are ln (protein abundance) in each replicate for bait protein (Spt6, indicated by ∗), histone proteins, RNAPII subunits, FACT, Paf1C, Spn1, and Spt5.

## Results

### Spt6 interaction with Spn1 contributes to its association with elongating RNAPII

Spt6 and Spn1 form a stable heterodimer within the RNAPII elongation complex (Fig. 1*A*) and ref (10). Multiple single amino acid substitutions within the binding regions of Spt6 and Spn1 have been previously identified and shown to disrupt their association without affecting their protein stabilities at nonpermissive temperatures (Fig. 1*B*) and see refs (13, 17, 19, 20). These and other studies (4) have demonstrated the broad significance of Spt6-Spn1 association in maintaining proper chromatin organization and gene transcription. However, our understanding of how Spt6-Spn1 interaction contributes to chromatin organization and gene transcription remains limited. To further investigate the importance of this interaction for gene transcription and chromatin organization, we first examined the necessity of the Spt6-Spn1 interaction for association with other members of the transcriptional machinery. Spt6-containing complexes were purified from strains harboring endogenously FLAG-tagged *SPT6* that was either wild-type or engineered to contain Spt6 or Spn1 mutants (*spt6-F249K*, *spn1-F267E*, and *spn1-R263D*) that disrupt the Spt6-Spn1 interaction upon shifting to non-permissive temperature (Fig. S1*A*). These complexes were then subjected to rigorous and quantitative mass spectrometric analysis. Principal Components Analysis (PCA) showed that the wild-type samples clustered together and were separated from the Spt6 and Spn1 mutants, which co-clustered together (Fig. S1*B*). Co-clustering of the Spt6 and Spn1 mutants in PC space suggests a similar impact of all three mutants on the Spt6 interactome. Further examination of all three mutants revealed large changes in protein-protein interactions with Spt6, including decreased interactions with RNAPII and RNAPII-associated elongation factors Paf1C, FACT, and Spt5 (Fig. 1, *C*–*F*). Most notable was the significant decrease in Spn1 association in all *spt6* and *spn1* mutants examined. These findings indicate that Spt6-Spn1 association is required for these chaperones to be stably part of the RNAPII elongation complex. Also of note was the decreased interaction with histones, which is likely a result of the Spt6 and Spn1 mutants being inappropriately released from actively transcribing RNAPII and/or the disruption of their ability to associate with their histone targets. Consistent with previous studies (14, 17, 19, 20), and as revealed in the hierarchical clustering shown in Figure 1*F*, the *spt6-F249K* mutant had the most severe impact on Spt6 interactions followed by the *spn1-F267E* and *spn1-R263D* mutants. Finally, STRING.db network analyses (21) of known protein-protein interactions for proteins with significantly decreased interactions with Spt6 in all three mutants highlighted the impact of these Spt6 and Spn1 blocking mutants on pathways associated with RNAPII and chromatin modifications (Fig. S1, *C*–*F*). A heatmap of all proteins that had decreased interactions with Spt6 relative to WT in at least one of the mutants is presented in Fig. S1*G*. Taken together, these data reveal that the interaction between Spt6 and Spn1 is important for proper association with the transcribing machinery and chromatin.

### Spt6-Spn1 association is required for proper nucleosome positioning within actively transcribed genes

**Figure 2:**
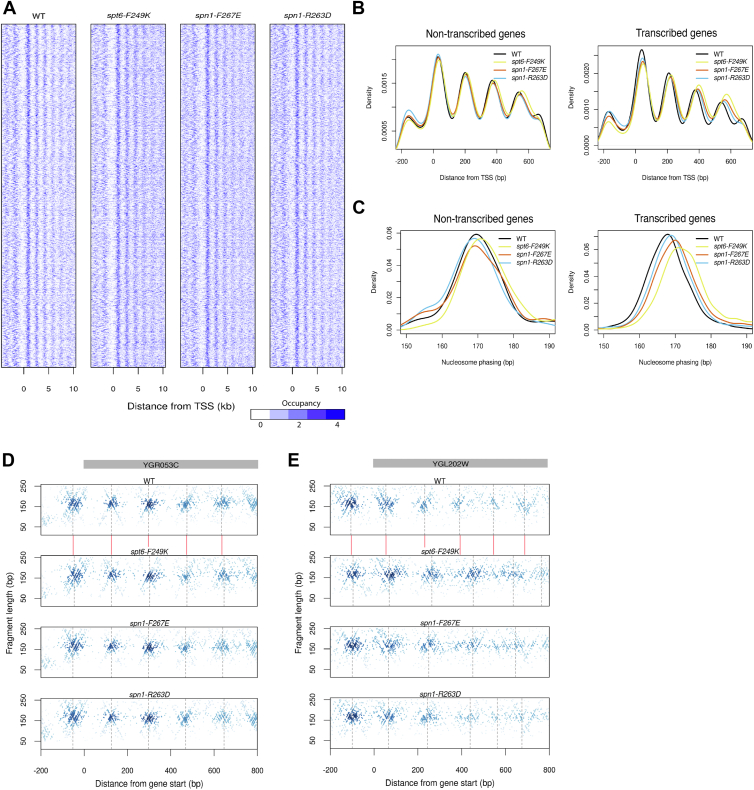
**Disruption of Spt6-Spn1 interaction results in increased nucleosome phasing in transcribed genes.***A*, heat maps of nucleosome profiles were generated by plotting the midpoints of the nucleosome-sized fragments surrounding the transcription start sites of 4649 non-overlapping genes with annotated transcription start sites (24). All panels are ordered by decreasing transcriptional activity. *B*, aggregated nucleosome profiles of genes with regularly phased nucleosomes grouped into non-transcribed genes (*left panel*) and transcribed genes (*right panel*). *C*, nucleosome phasing of non-transcribed genes (*left panel*) and transcribed genes (*right panel*) with regularly-phased nucleosomes. *D* and *E*, chromatin occupancy profiles for (*D*) *YGR053G*, a non-transcribed gene and (*E*) *YGL202W*, a transcribed gene. Fragment midpoints are plotted by fragment length and genomic position relative to ORF start. The shading of each point is determined by a 2D kernel density estimate of the surrounding points. Gene bodies are shown in *gray* on the *top*. *Grey dotted lines* denote the position of nucleosome dyads. *Red lines* between the WT plot and the *spt6-249K* mutant plot is to illustrate the relative changes to nucleosome positioning found in the *spt6* and *spn1* mutants when using the WT dyad midpoints as an anchor.

We next assessed how disruptions to Spt6-Spn1 interaction impact chromatin organization. To do so, we conducted genome-wide histone occupancy profiling to characterize the chromatin landscape within the different *spt6* and *spn1* mutant strains (22, 23). Briefly, chromatin was digested with MNase, after which protected DNA fragments were recovered and subjected to high-throughput paired-end sequencing. We focused on nucleosomal-sized fragments (∼150 bp) to assess the global chromatin structure in each of the mutants relative to WT. In Figure 2*A*, the midpoints of nucleosomal-sized fragments are oriented relative to the transcription start-site (TSS) (24) of 4649 non-overlapping genes, ordered by decreasing transcriptional activity. In all strains examined, we observed strong nucleosomal positioning with a canonical signal of a nucleosome-depleted region (NDR) and very well-positioned nucleosomes flanking the TSS and NDR. However, further examination of the mutant *spt6* and *spn1* profiles upon normalization relative to WT revealed a subtle but distinct downstream (*i.e.*, more 3′) shift of nucleosome positioning in each of the *spt6* and *spn1* mutants (Fig. S2). Similar 3′ shifts in nucleosome positioning in *spt6* and/or *spn1* mutants have also been reported by others (13, 25, 26).

The impact of the Spt6-Spn1 blocking mutants on nucleosome positioning compelled us to next quantify these changes more precisely and to determine if the nucleosome positioning changes observed in these mutants were restricted to actively transcribed genes or other regions (*e.g.*, non-transcribed genes and intergenic regions) as well, which might be observed if nucleosome positioning were altered as a result of defects in replication-dependent nucleosome deposition. To examine these unknowns, we inspected the nucleosome signal over the gene bodies in aggregate for non-transcribed and transcribed genes, and found that the nucleosome signal for non-transcribed genes was almost indistinguishable between the mutant and WT cells (Fig. 2*B*, left); however, for actively transcribed genes, we observed that the +2 and + 3 nucleosomes are progressively shifted further downstream from the promoter, suggesting that the Spt6-Spn1 interaction is important for maintaining precise nucleosome phasing (distance between nucleosome dyads) (Fig. 2*B*, right). To quantify this nucleosome phasing defect, we used an autocorrelation function (ACF) (27, 28) to find the optimal periodicity that best describes the nucleosome signal as shown in Figure 2*B*. The distance between neighboring nucleosome dyads in WT cells is typically 172 bp (27) and thus, the nucleosome signals at intervals of 172 bp should be highly correlated. In contrast, at half the distance between nucleosome dyads (86 bp), we would expect an anti-correlated signal. We defined nucleosome phasing as the distance between nucleosome dyads that maximizes the ACF value for the nucleosome signal and calculated nucleosome phasing for individual genes in all strains (Fig. 2*C*). The results confirmed that all three mutants had a transcription-dependent increase in nucleosome phasing, with *spt6-F249K* exhibiting the strongest increase of ∼10 bp. Interestingly, the severity of the nucleosome phasing defects among the mutants is consistent with the degree to which the mutations impair the Spt6-Spn1 interaction (Fig. 1) (17, 19). Finally, we plotted the midpoints of fragments recovered from MNase digestion according to their chromosomal positions for individual loci, categorizing them as either non-transcribed (Fig. 2*D*) or transcribed (Fig. 2*E*). On actively transcribed genes, a clear ∼10 bp rightward shift in nucleosome positioning is observed relative to the dyad axis for WT cells. Notably, this finding is fully consistent with previous studies that show the nucleosome spacing of highly transcribed genes in yeast is slightly shorter than those from lowly transcribed genes (29). A box plot comparison of the nucleosome phasing differences found between WT and the *spt6* and *spn1* mutants is shown in Fig. S3. Altogether, these data highlight the importance of Spt6-Spn1 interaction for maintaining the transcription-dependent shortening of nucleosome phasing.

### Spt6-Spn1-dependent change in nucleosome positioning results from a rotational shift of the nucleosomes

**Figure 3:**
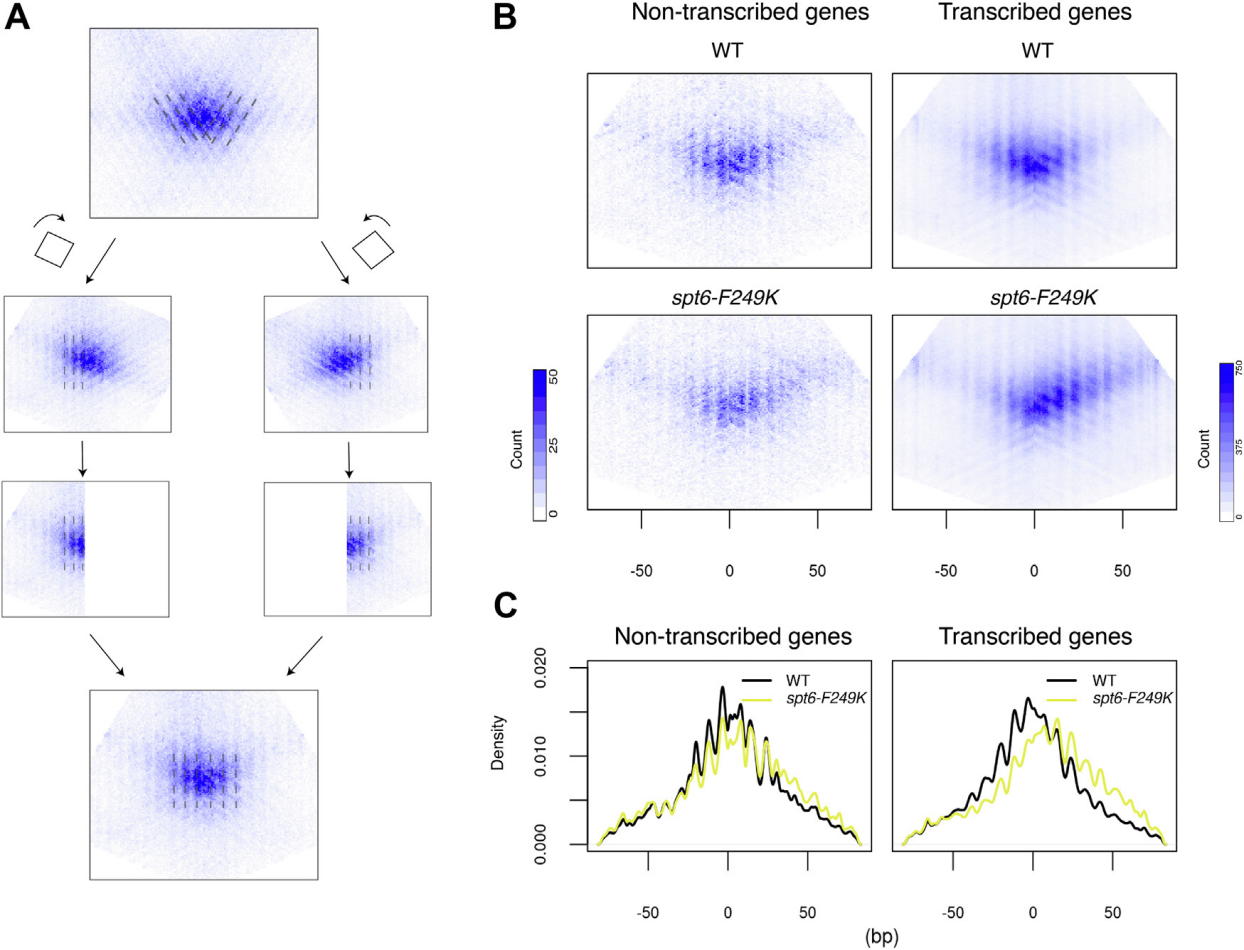
**Transcription-dependent increase in nucleosome phasing is associated with a 10 bp rotational shift of the nucleosomes.***A*, schematic describing the data processing steps for generating transformed nucleosome pile-ups. Nucleosome-sized reads (140–180 bp) around dyads of a group of nucleosomes were stacked up to generate the aggregate nucleosome profile. In the nucleosome profile, read midpoints form diagonal lines corresponding to changes in fragment length altering the midpoint of the protected fragment. The diagonal lines are spaced by multiples of 10 bp, reflecting alternative nucleosome dyad positions that can be attributed to the A/T dinucleotide repeat. To better visualize the pattern of nucleosome dyad positioning, the aggregate nucleosome profile was rotated 27 degrees clockwise, after which only the left half was retained. Similarly, the aggregate nucleosome profile was rotated 27 degrees counterclockwise, retaining the *right* half afterward. The two halves were joined to form the transformed nucleosome pile-up plots where alternative nucleosome dyad positions now align vertically. *B*, transformed nucleosome pile-up plots for the first three nucleosomes of non-transcribed genes (*left panels*) and transcribed genes (*right panels*). Nucleosomes are orientated relative to TSSs. *C*, density distribution of signals from the transformed nucleosome pile-up plots along the horizontals.

To further explore the mechanisms underlying the nucleosome positioning changes observed in the Spt6-Spn1 blocking mutants, we examined the distribution of MNase-protected fragments relative to the nucleosome dyad. A composite plot of nucleosome midpoints around 2000 high-confidence nucleosome dyads as defined by an orthogonal chemical cleavage approach (30) revealed an underlying structure in the positions of fragment midpoints relative to the chemically mapped dyad (Fig. 3*A*, top). Specifically, we observed that the midpoints lined up on diagonal lines (gray dotted line) corresponded to slight changes in fragment length altering the midpoint of the protected fragment. The positions appear to be spaced by multiples of 10 bp, which may be attributed to the A/T dinucleotide repeat found favored within the DNA sequence that wraps around histone octamers (31).

To better visualize these dyad positions, we transformed the composite plot by rotating the left half of the plot by 27° clockwise and the right half of the plot by 27° counterclockwise and merging the two halves (Fig. 3*A*, bottom) so that the dyad positions line up vertically. The original composite took into account the orientation of nucleosomes relative to TSSs. We made the transformed composite heatmaps for nucleosomes lying within the first 700 bp of gene bodies (Fig. 3*B*), grouped by their transcriptional activity, for both WT and mutant cells. For this analysis, we focused on *spt6-F249K* as it exhibits the most dramatic change in nucleosome positioning (Fig. 2). We found that in this mutant, the distribution of the nucleosome dyad is shifted downstream by 10 bp, where the central nucleosome dyad position now aligns with the next alternative A/T dinucleotide position (Fig. 3*C*). We believe that this represents a rotational change in the nucleosome; however, an alternative hypothesis is that this shift is dependent on an asymmetric sensitivity to MNase at the downstream (relative to transcription) edge of the nucleosome. To test this hypothesis, we examined the distribution of nucleosome-protected fragment edges—if the putative downstream rotational shift of the nucleosome dyad is due to asymmetric MNase sensitivity at the fragment edges, we would expect to see the nucleosome edge proximal to the promoters shifted downstream while the other nucleosome edge remained at its original position. We found that both nucleosome edges shift downstream by 10 bp, consistent with a rotational change of the nucleosome (Fig. S4). As with the defect in nucleosome phasing, the rotational shift of the nucleosome depends on transcriptional activity. The results suggest that nucleosomes can sample multiple favorable positions as they are assembled in the wake of transcriptional activity, and the Spt6-Spn1 association facilitates the establishment of proper nucleosome positioning to maintain nucleosome phasing.

### Spt6-Spn1 interaction regulates the distribution of nucleosomes and RNAPII across genes

**Figure 4:**
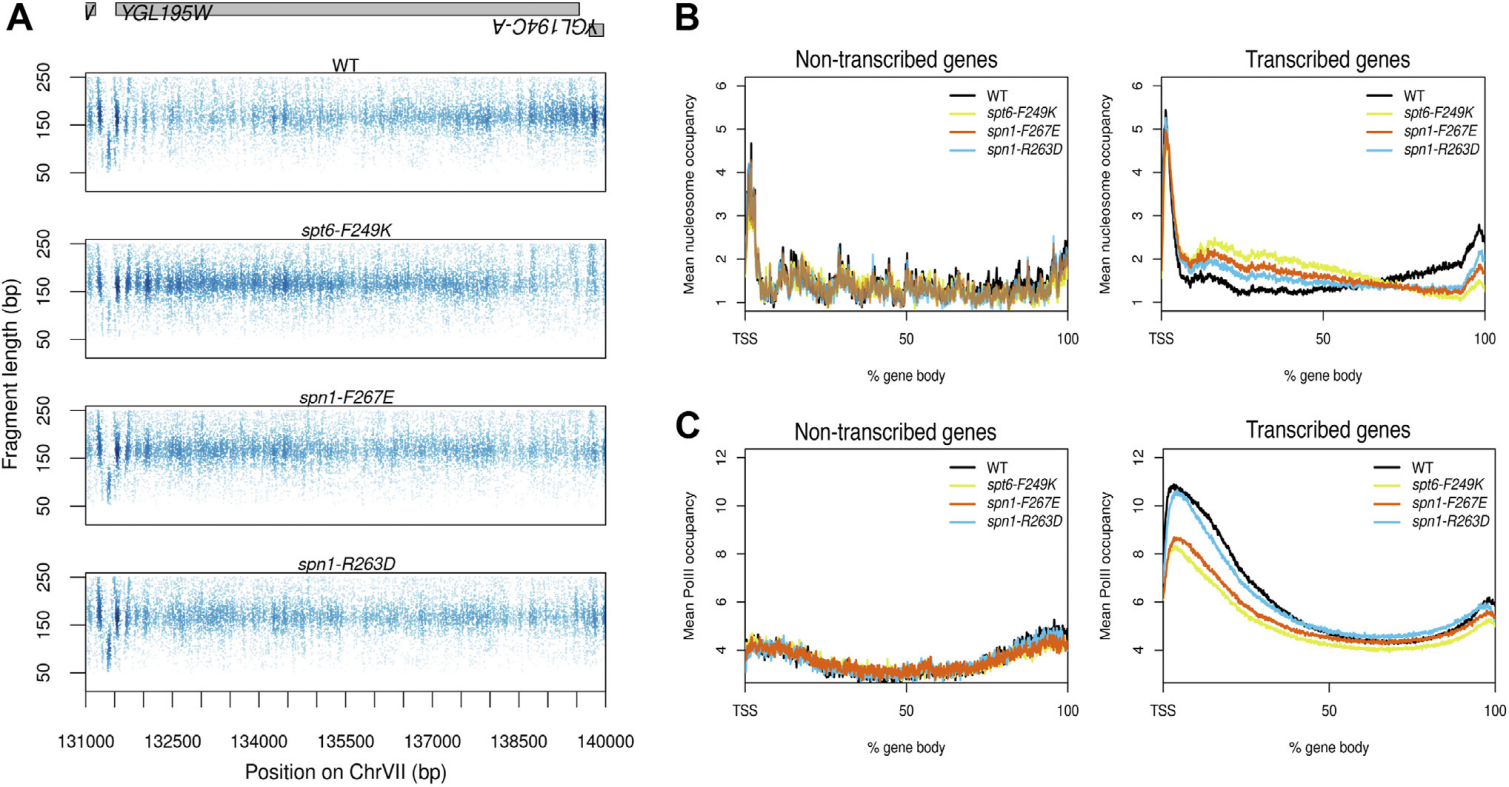
**Disruption of Spt6-Spn1 interaction causes a shift in the distribution of nucleosome and RNAPII occupancy across gene bodies.***A*, chromatin occupancy profile at a representative locus showing a shift in nucleosome density across the gene body. *B*, mean nucleosome occupancy across gene bodies of non-transcribed genes and transcribed genes. *C*, mean RNA polymerase II occupancy across gene bodies of non-transcribed genes and transcribed genes.

In addition to the transcription-dependent phasing and/or rotational defects observed in Spt6-Spn1 interaction mutants, we also observed a transcription-dependent shift in overall nucleosome density across gene bodies. While actively transcribed genes typically have well-positioned nucleosomes at the 5′ end of the gene, they also exhibit increased nucleosome occupancy (although not as well positioned) at the 3′ end of the gene (32). In Spt6-Spn1 interaction-blocking mutants, this accumulation of nucleosomes at the 3′ end of genes was reversed and they now accumulate at the 5′ end (Fig. 4*A*) and the severity of the change correlated with the mutant interaction phenotypes. This shift in nucleosome density, like the phasing and rotational changes was also transcription-dependent (Fig. 4*B*).

Well-positioned nucleosomes are thought to serve as a barrier to restrict transcription, yet during transcription, a combination of transcription elongation factors and chromatin remodelers promote the displacement of nucleosomes and facilitate RNAPII progression (33). Considering the dynamic relationship between nucleosomes and RNAPII, we sought to determine whether the change in nucleosome occupancy is accompanied by a change in RNAPII occupancy. We performed RNAPII ChIP-seq and found that both *spt6-F249K* and *spn1-F267* showed a flattening of RNAPII occupancy across the gene bodies with the occupancy of RNAPII being decreased at the 5′ end in the more severe *spt6* and *spn1* blocking mutants (Figs. 4*C* and S5). Additionally, the degree of RNAPII decrease was correlated with the degree to which nucleosome 5′ shifting increases (Fig. S6). While it is difficult to conclude if there is a causal relationship between the nucleosome and RNAPII occupancy changes observed, the results highlight the importance of Spt6-Spn1 association in maintaining the proper distribution of both nucleosomes and RNAPII across gene bodies.

### Impact of Spt6-Spn1 interaction on transcriptional regulation

Finally, we sought to determine how the nucleosome and RNAPII changes observed in our *spt6* and *spn1* mutants correlated with changes in global gene transcription and the production of 5′ antisense transcripts. We focused on 5′ antisense transcripts as our previous studies found these transcripts to commonly arise in mutants of Spt6 that prevent CKII-mediated phosphorylation, which leads to reduced Spt6-Spn1 association (17). Accordingly, we performed a stranded RNA sequencing (RNA-seq) analysis in our WT *spt6* and *spn1* mutant strains at permissive and restrictive temperatures. We first used DESeq2 to determine the differential RNA abundance between genes in the *spt6* and *spn1* mutants upon temperature shifting. As shown in the MA plots in Fig. S7*A*, our analyses found that the greatest degree of RNA change was observed with the *spt6-F249K*, followed by the *spn1-F267* and *spn1-R263D* mutants. These changes are consistent with previous findings (17) and correlate with the degree of phenotype associated with each of the three mutants. The three mutants also showed a high level of overlap, which would be expected for mutations impacting the same binding interface (Fig. S7, *B* and *C*). As expected from previous studies, *spt6* and *spn1* mutations resulted in both up- and down-regulation of gene expression that is associated with various cellular pathways including ribosomal and vacuole function based on GO analysis (Fig. S7*D*). Intriguingly, additional analyses of the RNA-seq data set for relationships with genomic features showed that genes down-regulated in the *spt6* and *spn1* mutants tend to have longer transcribed regions as compared to other genes; more highly transcribed genes also tended to be downregulated (Fig. S7, *E* and *F*). These findings are consistent with defects in transcription elongation, which are further reinforced by the altered distribution of RNAPII in these mutants.

Finally, we examined the *spt6-F249K*, *spn1-F267*, and *spn1-R263D* mutants for the presence of 5′ antisense transcripts. Using our previous data set as an anchor (17), we were able to identify the presence of 5′ antisense transcripts in the *spt6-F249K* and *spn1-F267* mutants (Fig. S7*G*). The *spn1-R263D* mutant, which shows the weakest phenotype among all of the mutants tested, trended upward for the presence of 5′ antisense transcripts but was not statistically significant compared to the wild type. Given the limited number of antisense transcripts observed in mutants that disrupt Spt6-Spn1 interaction, we asked if these genes harbor a unique chromatin signature that might explain why they undergo antisense transcription. However, examination of our MNase profiling data showed these antisense-producing genes had similar chromatin signatures and nucleosome changes found with most other transcribed genes (data not shown). We take these findings to surmise that while nucleosome positional changes are likely needed for antisense transcription to occur, it is the underlying DNA sequence at these genes that is required for such initiation events to occur.

## Discussion

In this report, we investigated the consequences of disrupting the Spt6-Spn1 interaction on its protein interaction network and on chromatin organization. Our findings show that Spt6 association with Spn1 is vital to the stability of Spt6 and Spn1 in the elongating RNAPII complex and that this disruption leads to defects in precise nucleosome positioning as well as in global distribution changes in nucleosome density and RNAPII distribution along transcribed genes. Although the mechanistic basis for this regulation is not known, it is likely a consequence of one or more activities associated with Spt6-Spn1—these include the ability of Spt6 to promote transcriptional elongation (rate and/or processivity) and to promote nucleosome reassembly in the wake of RNAPII elongation. Further studies will be needed to deconvolve the individual functions of Spt6-Spn1 in these processes.

An important observation made in this study is the finding that Spt6-Spn1 is required for the maintenance of precise nucleosome positioning along actively transcribed genes. Loss of Spt6-Spn1 interaction resulted in a rotational shift of about 10 bp along actively transcribed genes which ultimately led to increased nucleosomal phasing. These chromatin perturbations were specific for transcribed genes. While these differences in nucleosome positioning were dependent on transcription, we did not observe a direct correlation with transcriptional activity—rather any amount of transcription was sufficient to reset the nucleosome positions from those deposited by DNA replication. Together these results underscore a complex interplay among several factors—sequence dependence, transcription factor (TF) binding, DNA replication, and transcription—in establishing and maintaining chromatin architecture.

Studies show that Spt6 and other chaperones including FACT play important roles in resetting and maintaining nucleosome structure in the wake of RNAPII transcription (4, 13, 20, 34, 35). Our findings therefore support the important role of the Spt6-Spn1 heterodimer in ensuring proper resetting of nucleosomes to their prior state observed initially after replication. Intriguingly, a separate study by the Winston laboratory showed that reducing Spt6-Spn1 interaction also results in increased FACT association at genes (13). The authors proposed a model wherein Spt6-Spn1 and FACT levels at genes are carefully balanced for optimal function; in the case of Spt6-Spn1 loss, FACT levels increase as a compensation mechanism. Although our study did not examine the location of FACT in the specific *spt6* and *spn1* mutants employed here, we surmise that these mutants would also have resulted in increased FACT levels (and perhaps other chromatin-associated proteins) at genes. Given this, it is possible that some of the phenotypes reported herein with our *spt6* and *spn1* mutants that block their interaction are a result of increased FACT function. Further work will be needed to define the global chromatin changes that occur when Spt6 cannot associate with Spn1 and how these changes impact chromatin organization and gene transcription.

An important observation made in this study was the finding that disruption of Spt6-Spn1 interaction also altered the global distribution of nucleosomes towards the 5′ ends of actively transcribed genes (in addition to the 3′ rightward rotational shift described above; Fig. 4). Furthermore, the nucleosome occupancy 5′ shift was also correlated with reduced RNAPII levels (Figs. 4, S5 and S6). While the mechanistic basis for these alterations is unknown, such distribution changes with nucleosomes and RNAPII would be consistent with reduced initiation events and/or RNAPII elongation efficiency. Consistent with this idea, acute depletion of mammalian/yeast Spt6, or mutations in RNAPII that reduce elongation efficiency, also lead to reduced or altered RNAPII levels across active genes (15, 36, 37, 38, 39, 40). We conclude that some of the nucleosome and RNAPII changes are directly related to the impacts of not having Spt6 and Spn1 stably associated with RNAPII, which are known to promote RNAPII elongation in addition to its function as a histone chaperone.

Regarding cryptic transcription, our previous studies revealed a role for CKII-mediated phosphorylation of Spt6 in suppressing the production of 5′ antisense transcripts that were correlated with the enhancement of Spt6-Spn1 interaction (17). Notably, blocking or preventing this phosphorylation resulted in antisense or cryptic transcription from a subset of genes. Our studies confirmed the presence of these cryptic transcripts in our Spt6-Spn1 disrupting mutants, thereby further supporting the importance of CKII in maintaining proper Spt6-Spn1 function at genes. However, we note that the nucleosome occupancy and rotational shifting patterns we observed in our *spt6* and *spn1* mutants were found at all actively transcribed genes. Thus, while alterations in nucleosome occupancy and nucleosome positioning are important to the ability of such antisense transcripts to arise at these genes, other DNA sequence features are clearly required. This idea is fully supported by studies from the Winston lab, which found that many of the intragenic and genic sites of transcription that arise in mutants of *spt6* are associated with sequence elements that resemble gene promoters (15).

In conclusion, this work provides additional support for the importance of the Spt6-Spn1 interaction in maintaining proper nucleosome positioning and RNAPII levels at active genes. While distinct functions have been individually ascribed to Spt6 and Spn1, their heterodimer formation is clearly a necessity for their association with elongating RNAPII and histones. Reducing this association results in several chromatin and RNAPII changes at active genes and these effects are likely due to the inabilities of Spt6-Spn1 to promote transcription elongation and reassemble nucleosomes. In the future, it will be interesting to determine if the mammalian counterparts (SPT6 and IWS1) also form a heterodimer that is indispensable for their functions with RNAPII and chromatin.

## Experimental procedures

### Yeast strains and cellular growth

Wild-type (S288c), *spt6-F249K*, *spn1-R263D*, and *spn1-F267E* strains were engineered with a C-terminal 3X FLAG tag in the *SPT6* gene using standard yeast manipulation protocols (41). The integrations were confirmed by PCR and immunoblotting. The *spt6-F249K*, *spn1-R263D*, and *spn1-F267E* temperature-sensitive strains were grown in YPD to an OD_600_ of 0.6 at 30 °C. An equal volume of YPD medium pre-warmed to 44 °C was added, and the cultures were shifted to 37 °C for an additional 120 min prior to harvesting for all experiments. We note that our MNase studies revealed that the *spt6-F249K* strain harbors an additional chromosome XVI copy, which might be due to the need for a suppressor mutation for these cells to survive. Although the nature of the suppression is unknown, we note that the *SPN1* gene is on chromosome XVI, which might contribute to the improved growth of this strain. Despite the extra chromosomal copy, all *spt6* and *spn1* mutants examined showed similar trends with their nucleosome, RNA, and RNAPII alterations, suggesting this additional chromatin copy is not itself the cause of the phenotypes shown herein.

### Chromatin profiling by MNase-seq

MNase digestions were performed as described previously (22) and library preparations of the ideally digested samples (generating a nucleosomal ladder) were performed as previously described (27).

### RNA-seq and ChIP-seq

For both RNA-seq and ChIP-seq experiments, yeast strains were grown as described above and temperature shifts were performed. RNA extractions and stranded libraries were prepared as described previously (17). ChIPs were performed as described in Dronamraju *et al.* 2018 (17), with minor modifications. In the current set of experiments, after the temperature shift and before cell lysis and sonication, yeast cells were mixed with 1% *S. pombe* yeast cells. Following the spike-in of *S. pombe*, lysis, sonication, and immunoprecipitation were performed with total RNAPII antibody as described previously (17). Libraries were prepared using standard methods and were sequenced.

### Bioinformatics analyses

MNase-seq reads were aligned to the sacCer3/R61 version of the *S.cerevisiae* genome using Bowtie 0.12.7 (42) in paired-end mode using the following Bowtie parameters: -n 2 -l 20 -m 1. Biological replicates were merged. To normalize across strains, the fewest number of reads for each fragment size (20–250 bp) was identified and used as the subsampling depth. Nucleosome-sized reads were defined as those with lengths between 140 bp and 180 bp. All analyses were performed in R (R Core Team 2024).

#### ChIP-seq data processing

Sequencing adapters were trimmed using cutadapt requiring a minimum trimmed length of at least 36 bp. After trimming, reads were filtered for quality using FASTX-Toolkit fastq_quality_filter. Potential PCR duplicates were then removed by limiting reads with the same sequence to a maximum of five copies. After deduplication, reads were aligned to the reference yeast genome (sacCer3) combined with the *S. pombe* reference (Ensembl EF2) to account for spike-ins using STAR (v2.5.2 b). Following alignment, SAMtools and BEDTools were used to filter for primary alignments and generate normalized bigWigs. RNA-seq reads were processed identically to that of ChIP-seq with the exception that PCR duplicates were not removed and the reads were aligned only to the sacCer3 reference genome.

For ChIP-seq and RNA-seq, biological replicates were merged and ChIP-seq reads were subsampled to the same read depth across strains. We arbitrarily defined transcribed genes as those with log2 (RPKM + 1) ≥ 0.5. The R package DESeq2 (43) was used to find differentially expressed genes.

### Proteomic analyses

#### Protein preparation

For proteomic studies, Spt6 3XFLAG, Spt6 3XFLAG *spn1-R263D*, Spt6 3XFLAG *spt6-F249K*, and Spt6 3XFLAG *spn1-F267E* were grown in 6L cultures to an OD_600_ of 3.0. Four biological replicates of each strain were prepared for each strain. Cells were harvested and washed with HPLC water. The resulting pellet was frozen overnight at −80 °C. The thawed pellet was put dropwise *via* a syringe into a prechilled mortar containing liquid nitrogen. A pestle was used to further grind the sample into a consistency similar to “Dippin Dots” (as previously described in (34)). The sample was moved into a prechilled Retsch Cryomill zirconium oxide vessel with impact ball. Lysis was performed *via* six cycles for 3 min each at 15 Hz. The lysed powder was resuspended in lysis buffer (40mM HEPES-KOH, pH 7.5; 10% glycerol; 350mM NaCl; 0.1% Tween-20) with 1 mM sodium orthovanadate and 1X protease inhibitor cocktail. Lysed cell suspension was placed on a stir plate at room temperature for 10 min with 100 units of DNase I and 10 μl of 30 mg/ml heparin. After this treatment, the sample was clarified by centrifugation at 4 °C for 1 h at 13,000 rpm. The resulting supernatant was incubated overnight at 4 °C with 500 μl of washed FLAG agarose resin slurry on a stir plate. The next morning the supernatant/resin mix was applied to a Bio Rad prep column and allowed to drain by gravity. The resin was washed on the column with 100 ml of the lysis buffer described above. Competitive elution was completed using FLAG peptide and lysis buffer. Four elutions were collected from each prep. A portion of these elutions were prepped for visualization on silver-stained gel and TCA precipitation was performed on a portion of the remaining elution for subsequent Nano-LC-MS/MS analysis. We note that mock purifications isolated in the same purification conditions or in lower salt conditions (150mM NaCl) to define non-specific background proteins that are isolated with the affinity resin in a non-tagged parental background have been previously reported (34, 40, 44, 45).

#### Digestion and peptide preparation

TCA precipitated pellets were resuspended in 8M urea. Reduction and alkylation were performed with a final concentration of 5 mM TCEP (Tris (2-carboxyethyl)phosphine hydrochloride) and 10 mM CAM (chloroacetamide). Trypsin/LysC (Promega) mix was added to the samples at a ratio of 1:25. After allowing them to digest for 4 h at 35 °C, the 8M urea concentration was reduced to 2M to allow for trypsin digest overnight. After quenching digest, the samples were dried in a speedvac and desalted on a Waters C18 column. Dried samples were resuspended in 25 μl 100 mM TEAB for labeling with Thermo Fisher Scientific TMT 16 plex. The labeled samples were pooled into one tube and dried *via* speedvac. The samples were resuspended in 0.1% TFA (trifluoroacetic acid) and then fractionated *via* High pH Fractionation Columns. The eight fractions were dried in a speedvac and resuspended in 0.1% formic acid for LC/MS.

#### LC-MS/MS

Nano-LC-MS/MS analyses were performed on an EASY-nLC HPLC system (SCR: 014,993, Thermo Fisher Scientific) coupled to an Orbitrap Fusion Lumos mass spectrometer (Thermo Fisher Scientific). One-fourth of each of the eight fractions was loaded onto a reversed-phase EasySpray C18 column (2 μm, 100 Å, 75 μm × 25 cm, Thermo Scientific Cat No: ES902A) at 400 nl/min. Peptides were eluted from 4 to 28% with mobile phase B (mobile phases A: 0.1% FA, water; B: 0.1% FA, 80% acetonitrile) over 170 min; 28 to 80% B over 5 min; and dropping from 50-10% B over the final 1 min. The mass spectrometer was operated in positive ion mode with a 4 s cycle time data-dependent acquisition method with advanced peak determination and Easy-IC (internal calibrant). Precursor scans (m/z 400–1600) were done with an orbitrap resolution of 120,000, RF lens% 30, maximum inject time auto, standard ACG target, including charges of two to seven for fragmentation with 30 s dynamic exclusion, 200% normalized ACG target and dynamic maximum IT. The data were recorded using Thermo Fisher Scientific Excalibur software.

#### Proteome Analysis

Resulting RAW files were analyzed using Proteome Discoverer 2.5 (Thermo Fisher Scientific), with a *Saccharomyces cerevisiae* UniProt FASTA plus common contaminants. Quantification methods utilized TMT isotopic impurity levels available from Thermo Fisher Scientific. SEQUEST HT searches were conducted with a maximum number of three missed cleavages, precursor mass tolerance of 20 ppm; and a fragment mass tolerance of 0.5 Da. Static modifications used for the search were, 1) carbamidomethylation on cysteine (C) residues; 2) TMTpro label on lysine (K) residues and the N-termini of peptides. Dynamic modifications used for the search were oxidation of methionines, phosphorylation of S, T, Y residues, and acetylation, Met-loss or Met-loss plus acetylation of protein N-termini. Percolator False Discovery Rate was set to a strict setting of 0.1 and a relaxed setting of 0.05. For the Proteome Discoverer consensus workflow, isobaric impurities corrections were turned on, the reporter ion co-isolation threshold was set to 50%, and the average signal to noise threshold was set at 5. All peptides were used for protein roll-up, but modified peptides were excluded for pairwise ratio testing. Differential expression of the protein groups was assessed by ANOVA (individual proteins) calculated *p*-values. Analyzed data from Proteome Discoverer was exported and further analyzed in RStudio (R Studio for Mac, version 2023.9.0.463) (46) and browser based ClustVis (47). Volcano plots were created using ggplot2 (46). Heat map, clustering and principal component analyses were performed in ClustVis (47). STRING.db network analysis was completed in RStudio using the STRING.db and STRINGutils R packages (48).

## Data availability

MNase profiling, RNA-seq and ChIP-seq data sets have been deposited to the Gene Expression Omnibus under GEO accession GSE284959. Proteomics data have been deposited with ProteomeXchange repository and are available under project accession: MSV000094759.

## Supporting information

This article contains supporting information (17, 21, 46).

## Conflict of interest

The authors declare the following financial interests/personal relationships which may be considered as potential competing interests.

BDS is a co-founder and board member of EpiCypher, Inc. BDS is an Editorial Associate Editor for *Journal of Biological Chemistry* and was not involved in the editorial review or the decision to publish this article.

## References

[1] Gurard-Levin Z.A., Quivy J.P., Almouzni G. (2014). Histone chaperones: assisting histone traffic and nucleosome dynamics. Annu. Rev. Biochem..

[2] Pardal A.J., Fernandes-Duarte F., Bowman A.J. (2019). The histone chaperoning pathway: from ribosome to nucleosome. Essays Biochem..

[3] Ray-Gallet D., Almouzni G. (2022). H3-H4 histone chaperones and cancer. Curr. Opin. Genet. Dev..

[4] Miller C.L.W., Warner J.L., Winston F. (2023). Insights into Spt6: a histone chaperone that functions in transcription, DNA replication, and genome stability. Trends Genet..

[5] Aoi Y., Shilatifard A. (2023). Transcriptional elongation control in developmental gene expression, aging, and disease. Mol. Cell.

[6] Robert F., Jeronimo C. (2023). Transcription-coupled nucleosome assembly. Trends Biochem. Sci..

[7] Ellison M.A., Namjilsuren S., Shirra M.K., Blacksmith M.S., Schusteff R.A., Kerr E.M. (2023). Spt6 directly interacts with Cdc73 and is required for Paf1 complex occupancy at active genes in Saccharomyces cerevisiae. Nucleic Acids Res..

[8] Vos S.M., Farnung L., Boehning M., Wigge C., Linden A., Urlaub H. (2018). Structure of activated transcription complex Pol II-DSIF-PAF-SPT6. Nature.

[9] Vos S.M., Farnung L., Linden A., Urlaub H., Cramer P. (2020). Structure of complete Pol II-DSIF-PAF-SPT6 transcription complex reveals RTF1 allosteric activation. Nat. Struct. Mol. Biol..

[10] Ehara H., Kujirai T., Shirouzu M., Kurumizaka H., Sekine S.I. (2022). Structural basis of nucleosome disassembly and reassembly by RNAPII elongation complex with FACT. Science.

[11] Li S., Almeida A.R., Radebaugh C.A., Zhang L., Chen X., Huang L. (2018). The elongation factor Spn1 is a multi-functional chromatin binding protein. Nucleic Acids Res..

[12] Li S., Edwards G., Radebaugh C.A., Luger K., Stargell L.A. (2022). Spn1 and its dynamic interactions with Spt6, histones and nucleosomes. J. Mol. Biol..

[13] Viktorovskaya O., Chuang J., Jain D., Reim N.I., Lopez-Rivera F., Murawska M. (2021). Essential histone chaperones collaborate to regulate transcription and chromatin integrity. Genes Dev..

[14] Lopez-Rivera F., Chuang J., Spatt D., Gopalakrishnan R., Winston F. (2022). Suppressor mutations that make the essential transcription factor Spn1/Iws1 dispensable in Saccharomyces cerevisiae. Genetics.

[15] Doris S.M., Chuang J., Viktorovskaya O., Murawska M., Spatt D., Churchman L.S. (2018). Spt6 is required for the fidelity of promoter selection. Mol. Cell.

[16] Reim N.I., Chuang J., Jain D., Alver B.H., Park P.J., Winston F. (2020). The conserved elongation factor Spn1 is required for normal transcription, histone modifications, and splicing in Saccharomyces cerevisiae. Nucleic Acids Res..

[17] Dronamraju R., Kerschner J.L., Peck S.A., Hepperla A.J., Adams A.T., Hughes K.D. (2018). Casein kinase II phosphorylation of Spt6 enforces transcriptional fidelity by maintaining spn1-spt6 interaction. Cell Rep..

[18] Gouot E., Bhat W., Rufiange A., Fournier E., Paquet E., Nourani A. (2018). Casein kinase 2 mediated phosphorylation of Spt6 modulates histone dynamics and regulates spurious transcription. Nucleic Acids Res..

[19] McDonald S.M., Close D., Xin H., Formosa T., Hill C.P. (2010). Structure and biological importance of the Spn1-Spt6 interaction, and its regulatory role in nucleosome binding. Mol. Cell.

[20] McCullough L., Connell Z., Petersen C., Formosa T. (2015). The abundant histone chaperones Spt6 and FACT collaborate to assemble, inspect, and maintain chromatin structure in Saccharomyces cerevisiae. Genetics.

[21] Szklarczyk D., Franceschini A., Wyder S., Forslund K., Heller D., Huerta-Cepas J. (2015). STRING v10: protein-protein interaction networks, integrated over the tree of life. Nucleic Acids Res..

[22] Belsky J.A., MacAlpine H.K., Lubelsky Y., Hartemink A.J., MacAlpine D.M. (2015). Genome-wide chromatin footprinting reveals changes in replication origin architecture induced by pre-RC assembly. Genes Dev..

[23] Henikoff J.G., Belsky J.A., Krassovsky K., MacAlpine D.M., Henikoff S. (2011). Epigenome characterization at single base-pair resolution. Proc. Natl. Acad. Sci. U. S. A..

[24] Park D., Morris A.R., Battenhouse A., Iyer V.R. (2014). Simultaneous mapping of transcript ends at single-nucleotide resolution and identification of widespread promoter-associated non-coding RNA governed by TATA elements. Nucleic Acids Res..

[25] Mayer A., Lidschreiber M., Siebert M., Leike K., Soding J., Cramer P. (2010). Uniform transitions of the general RNA polymerase II transcription complex. Nat. Struct. Mol. Biol..

[26] Tonsager A.J., Zukowski A., Radebaugh C.A., Weirich A., Stargell L.A., Ramachandran S. (2025). The histone chaperone Spn1 preserves chromatin protections at promoters and nucleosome positioning in open reading frames. bioRxiv.

[27] Gutierrez M.P., MacAlpine H.K., MacAlpine D.M. (2019). Nascent chromatin occupancy profiling reveals locus- and factor-specific chromatin maturation dynamics behind the DNA replication fork. Genome Res..

[28] Chen B., MacAlpine H.K., Hartemink A.J., MacAlpine D.M. (2023). Spatiotemporal kinetics of CAF-1-dependent chromatin maturation ensures transcription fidelity during S-phase. Genome Res..

[29] Chereji R.V., Morozov A.V. (2015). Functional roles of nucleosome stability and dynamics. Brief. Funct. Genomics.

[30] Brogaard K., Xi L., Wang J.P., Widom J. (2012). A map of nucleosome positions in yeast at base-pair resolution. Nature.

[31] Segal E., Fondufe-Mittendorf Y., Chen L., Thastrom A., Field Y., Moore I.K. (2006). A genomic code for nucleosome positioning. Nature.

[32] Lee C.K., Shibata Y., Rao B., Strahl B.D., Lieb J.D. (2004). Evidence for nucleosome depletion at active regulatory regions genome-wide. Nat. Genet..

[33] Hughes A.L., Jin Y., Rando O.J., Struhl K. (2012). A functional evolutionary approach to identify determinants of nucleosome positioning: a unifying model for establishing the genome-wide pattern. Mol. Cell.

[34] Victorino J.F., Fox M.J., Smith-Kinnaman W.R., Peck Justice S.A., Burriss K.H., Boyd A.K. (2020). RNA Polymerase II CTD phosphatase Rtr1 fine-tunes transcription termination. PLoS Genet..

[35] Formosa T., Winston F. (2020). The role of FACT in managing chromatin: disruption, assembly, or repair?. Nucleic Acids Res..

[36] Malik I., Qiu C., Snavely T., Kaplan C.D. (2017). Wide-ranging and unexpected consequences of altered Pol II catalytic activity in vivo. Nucleic Acids Res..

[37] Zhang L., Fletcher A.G., Cheung V., Winston F., Stargell L.A. (2008). Spn1 regulates the recruitment of Spt6 and the Swi/Snf complex during transcriptional activation by RNA polymerase II. Mol. Cell Biol..

[38] Narain A., Bhandare P., Adhikari B., Backes S., Eilers M., Dolken L. (2021). Targeted protein degradation reveals a direct role of SPT6 in RNAPII elongation and termination. Mol. Cell.

[39] Aoi Y., Shah A.P., Ganesan S., Soliman S.H.A., Cho B.K., Goo Y.A. (2022). SPT6 functions in transcriptional pause/release via PAF1C recruitment. Mol. Cell.

[40] Dronamraju R., Hepperla A.J., Shibata Y., Adams A.T., Magnuson T., Davis I.J. (2018). Spt6 association with RNA polymerase II directs mRNA turnover during transcription. Mol. Cell.

[41] Janke C., Magiera M.M., Rathfelder N., Taxis C., Reber S., Maekawa H. (2004). A versatile toolbox for PCR-based tagging of yeast genes: new fluorescent proteins, more markers and promoter substitution cassettes. Yeast.

[42] Langmead B., Trapnell C., Pop M., Salzberg S.L. (2009). Ultrafast and memory-efficient alignment of short DNA sequences to the human genome. Genome Biol..

[43] Love M.I., Huber W., Anders S. (2014). Moderated estimation of fold change and dispersion for RNA-seq data with DESeq2. Genome Biol..

[44] Bedard L.G., Dronamraju R., Kerschner J.L., Hunter G.O., Axley E.D., Boyd A.K. (2016). Quantitative analysis of dynamic protein interactions during transcription reveals a role for casein kinase II in polymerase-associated factor (PAF) complex phosphorylation and regulation of histone H2B monoubiquitylation. J. Biol. Chem..

[45] Mosley A.L., Hunter G.O., Sardiu M.E., Smolle M., Workman J.L., Florens L. (2013). Quantitative proteomics demonstrates that the RNA polymerase II subunits Rpb4 and Rpb7 dissociate during transcriptional elongation. Mol. Cell Proteomics.

[46] Wickham H., Navarro D., Lin T. (2016). Ggplot2: Elegant Graphics for Data Analysis.

[47] Metsalu T., Vilo J. (2015). ClustVis: a web tool for visualizing clustering of multivariate data using Principal Component Analysis and heatmap. Nucleic Acids Res..

[48] Szklarczyk D., Gable A.L., Nastou K.C., Lyon D., Kirsch R., Pyysalo S. (2021). The STRING database in 2021: customizable protein-protein networks, and functional characterization of user-uploaded gene/measurement sets. Nucleic Acids Res..

