# Supplementary material for: Spt6-Spn1 interaction is required for RNA polymerase II association and precise nucleosome positioning along transcribed genes
Source: J Biol Chem. 2025 Mar 22;301(5):108436. doi: 10.1016/j.jbc.2025.108436 (PMC12053661; doi:10.1016/j.jbc.2025.108436)
Supplement: Supporting Information 2.24.25 [file mmc2.docx]

Supporting Information for

**Spt6-Spn1 interaction is required for RNA Polymerase II association and precise nucleosome positioning along transcribed genes**

Boning Chen^1^, Raghuvar Dronamraju^2^, W.R. Smith-Kinnaman^3^, Sarah A. Peck Justice^4^, Austin J. Hepperla^5^, Heather K MacAlpine^1^, Jeremy M. Simon^6,7^, Amber L. Mosley^3^, David M MacAlpine^1^* & Brian D. Strahl^2,5^*

^1^Department of Pharmacology and Cancer Biology, Duke University Medical Center, Durham, NC 27710.

^2^Department of Biochemistry and Biophysics, University of North Carolina, Chapel Hill, NC 27599.

^3^Indiana University School of Medicine, Department of Biochemistry and Molecular Biology, Indianapolis, IN 46202.

^4^Department of Biology, Marian University, Indianapolis, IN 46222.

^5^ Bioinformatics and Analytics Research Collaborative (BARC), University of North Carolina at Chapel Hill, Chapel Hill, NC, 27599.

^6^Department of Data Science, Dana-Farber Cancer Institute, Boston, Massachusetts 02115

^7^Department of Biostatistics, Harvard T.H. Chan School of Public Health, Cambridge, Massachusetts 02115

* To whom correspondence should be addressed: [david.macalpine@duke.edu](mailto:david.macalpine@duke.edu) and [brian_strahl@med.unc.edu](mailto:brian_strahl@med.unc.edu)

Supplementary Figures S1-S6

Supplementary Table S1 (provided in separate Excel file)

References

**
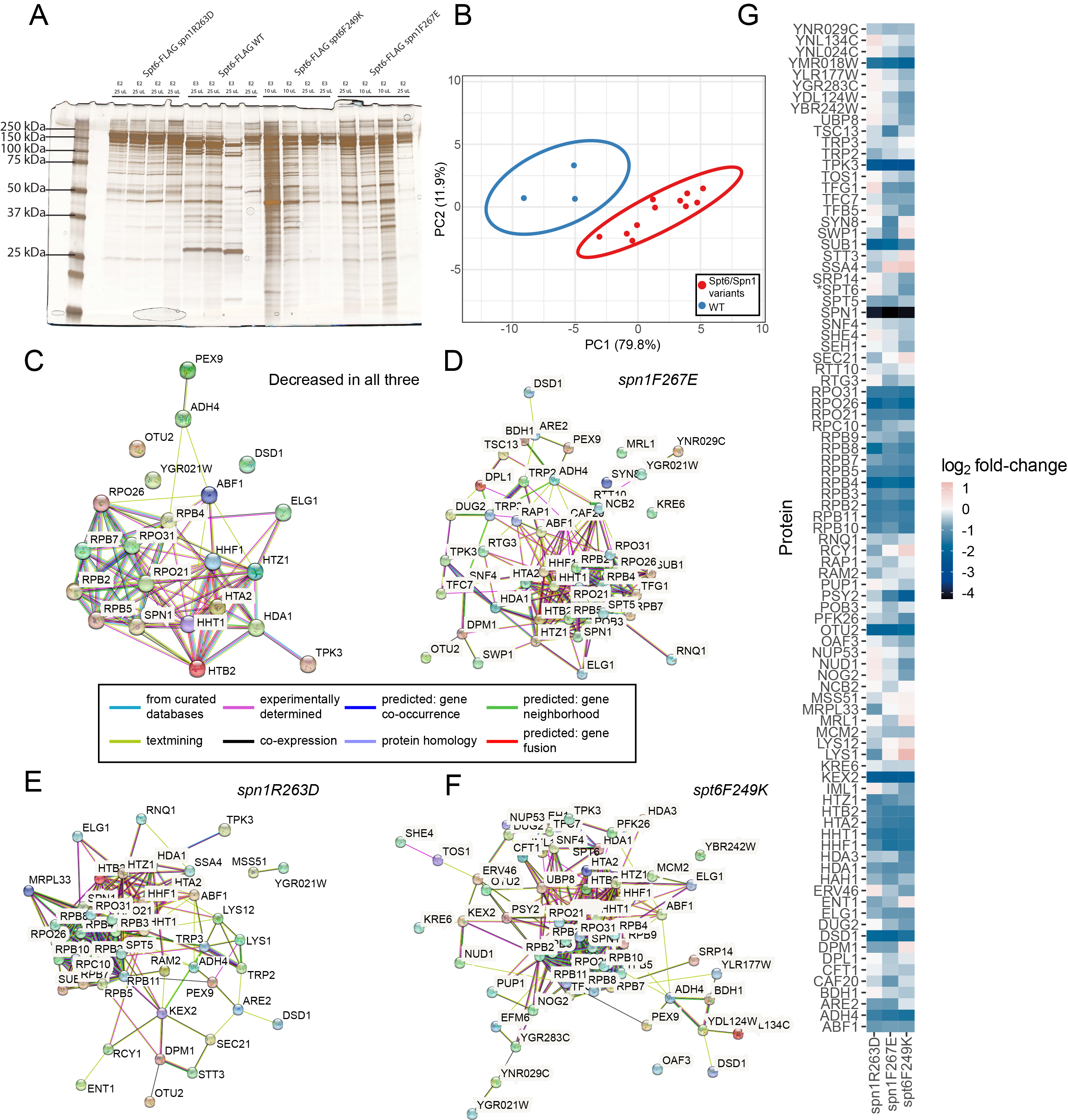
**

**Supplementary Figure 1. Protein interaction analysis defines the dependency of Spt6-Spn1 association for RNAPII and histone interaction.** A) Silver-stained gel showing purifications of Spt6-3XFLAG WT, *spt6* and *spn1* mutants that were used for mass spectrometric analysis. B) Principal Component Analysis of replicates of Spt6-3XFLAG in WT and Spt6 or Spn1 mutants that prevent their interaction. PCA was performed using ClustVis to compare each replicate sample from Spt6-3XFLAG WT (n=3), Spt6-3XFLAG *spn1-F267E* (n=3), Spt6-3XFLAG *spn1-R263D* (n=4), and Spt6-3XFLAG *spt6-F249K* (n=4). WT samples are shown in blue, Spt6/Spn1 variants in red. C-E) STRING.db network analysis (1) of known protein-protein interactions for proteins with significantly decreased interactions with Spt6 in C) all three mutants (n=22), D) *spn1-F267E* (n=47), E) *spn1-R263D* (n=45), and F) *spt6-F249K* (n=60). G) Heatmap (ggplot2)(2) of log_2_ fold-change of mutant vs. wildtype for proteins that were significantly (p-value < 0.05) decreasing in at least one of the three mutants.


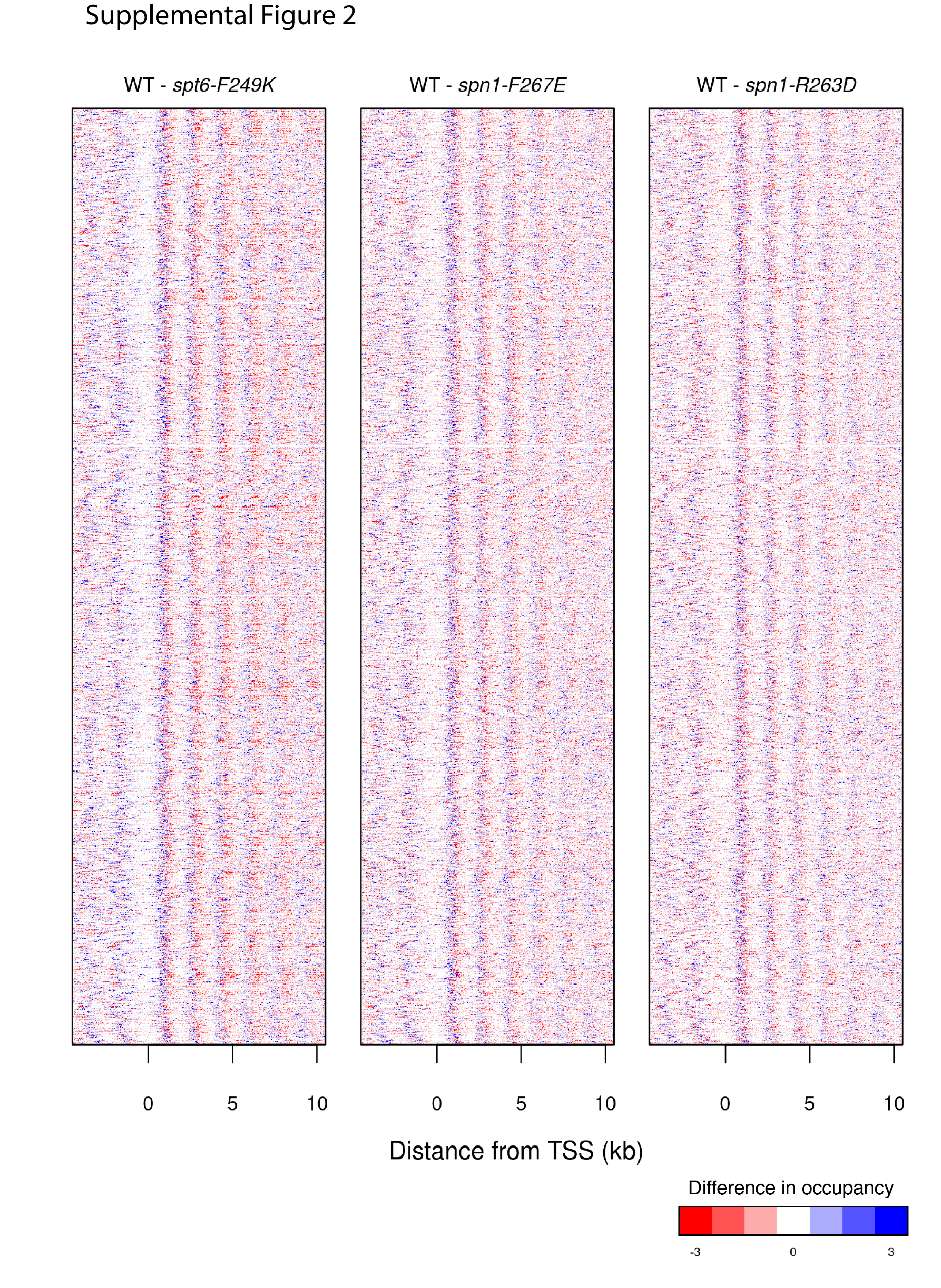


**Supplementary Figure 2. Spt6-Spn1 interaction blocking mutants exhibit a rightward 3’ shift of nucleosome positioning.** Heatmaps depicting differential nucleosome signals between WT and the mutants for the identical gene set as shown in Figure 2A.

**
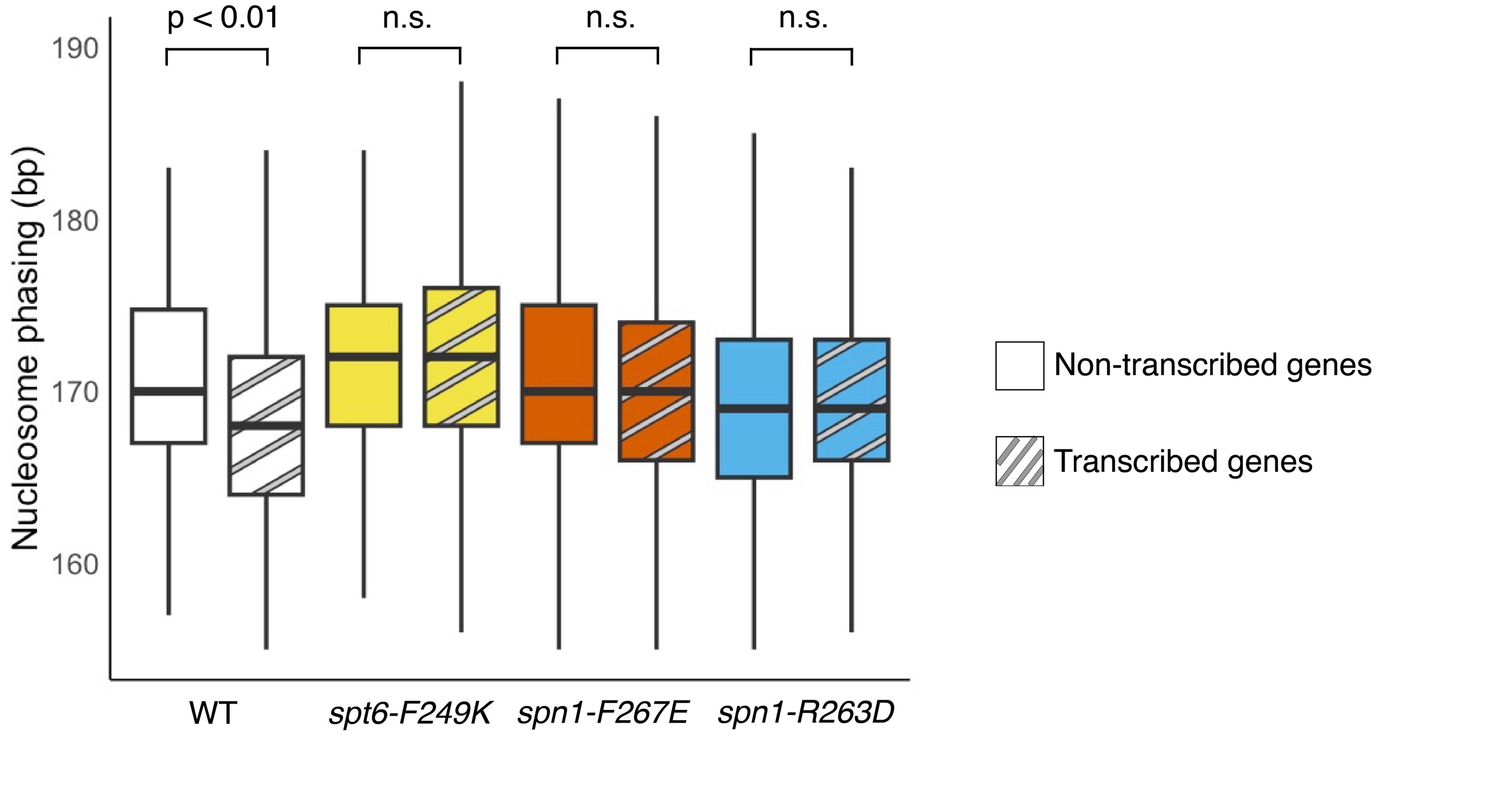
**

**Supplementary Figure 3.** **Spt6-spn1 mutations disrupt transcription-dependent decrease in nucleosome phasing.** Box plot comparing nucleosome phasing between transcribed genes and non-transcribed genes in all strains.

**
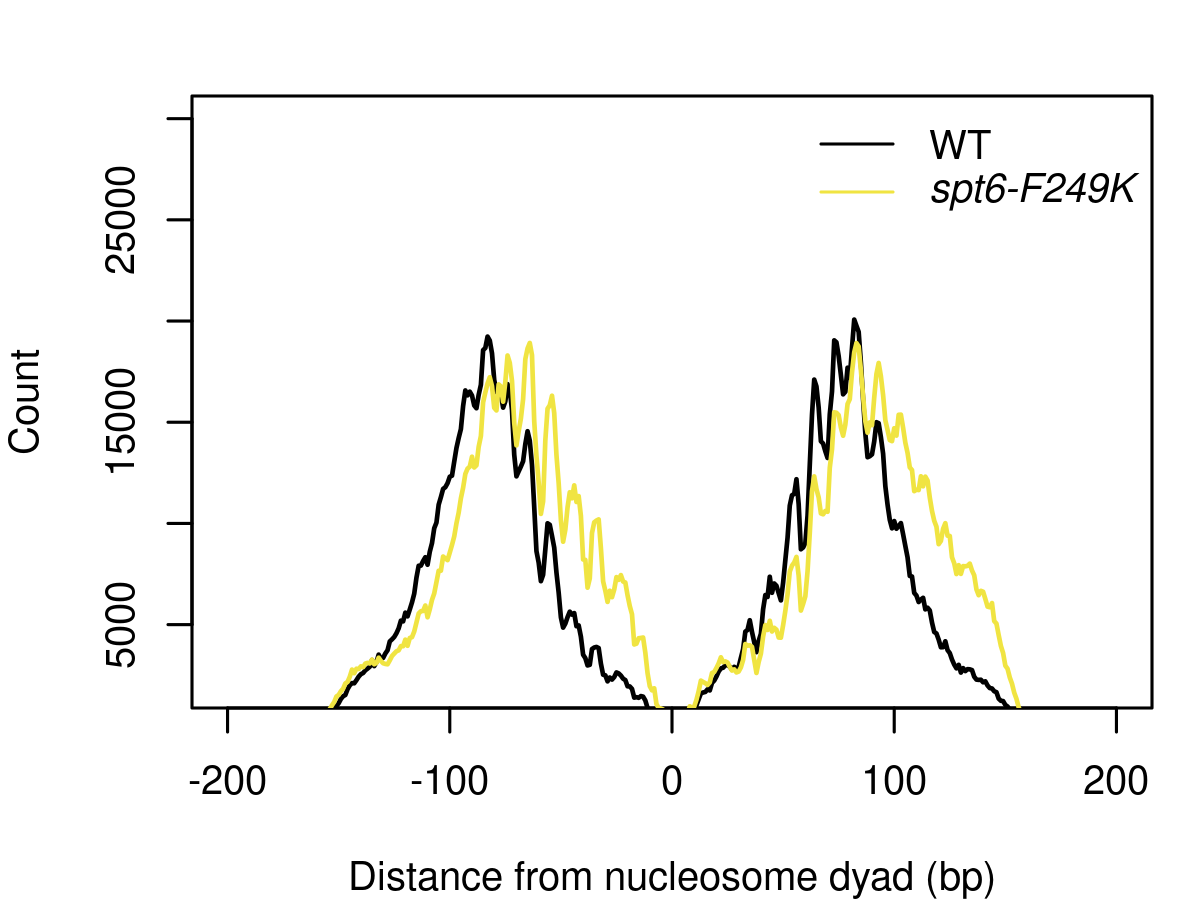
**

**Supplementary Figure 4. Spt6-Spn1 interaction blocking mutations lead to a 10bp rotational shift of nucleosome dyads.** Histogram of fragment ends surrounding nucleosome dyads shown in Figure 3B for WT (black) and *spt6-F249K* (yellow). Fragment ends are orientated relative to TSSs—left peak corresponds to fragment ends proximal to TSSs while the right peak corresponds to fragment ends distal to TSSs. The coordinated shift in nucleosome ends is consistent with a rotational shift of the dyad and not differential sensitivity of the ends to MNase.


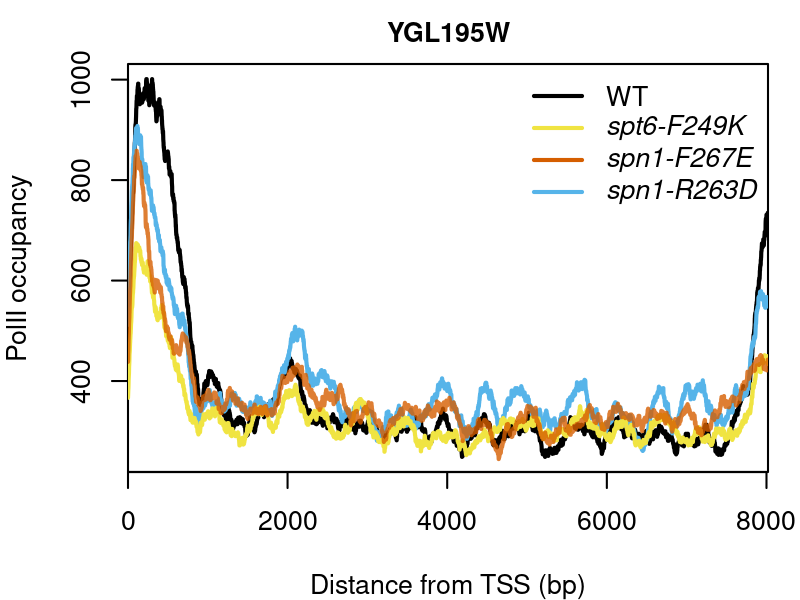


**Supplementary Figure 5. Disruption of Spt6-Spn1 interaction leads to decreased RNA polymerase II level, particularly at the 5’ end of gene bodies**. RNA polymerase II occupancy across the gene body of YGL195W.

**
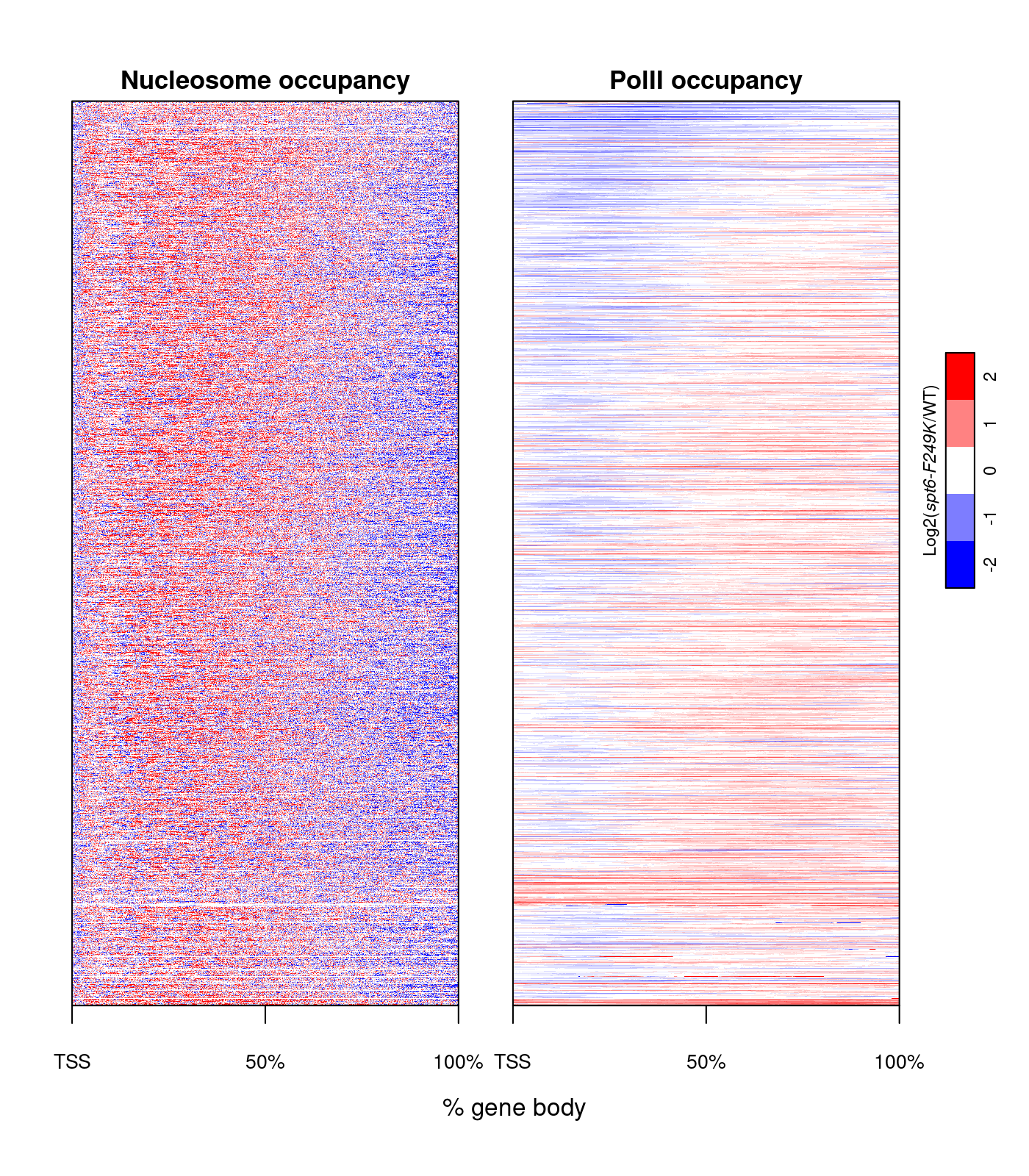
**

**Supplementary Figure 6. Spt6-Spn1 mutation induced changes in nucleosome occupancy and RNA Pol II occupancy are correlated with each other**. Heatmap showing log2 fold change in nucleosome occupancy (left) and RNA Pol II occupancy (right) between *spt6-F249K* and WT cells across the gene bodies of 3,427 genes longer than 1kb, ordered by decreasing transcriptional activities.

**
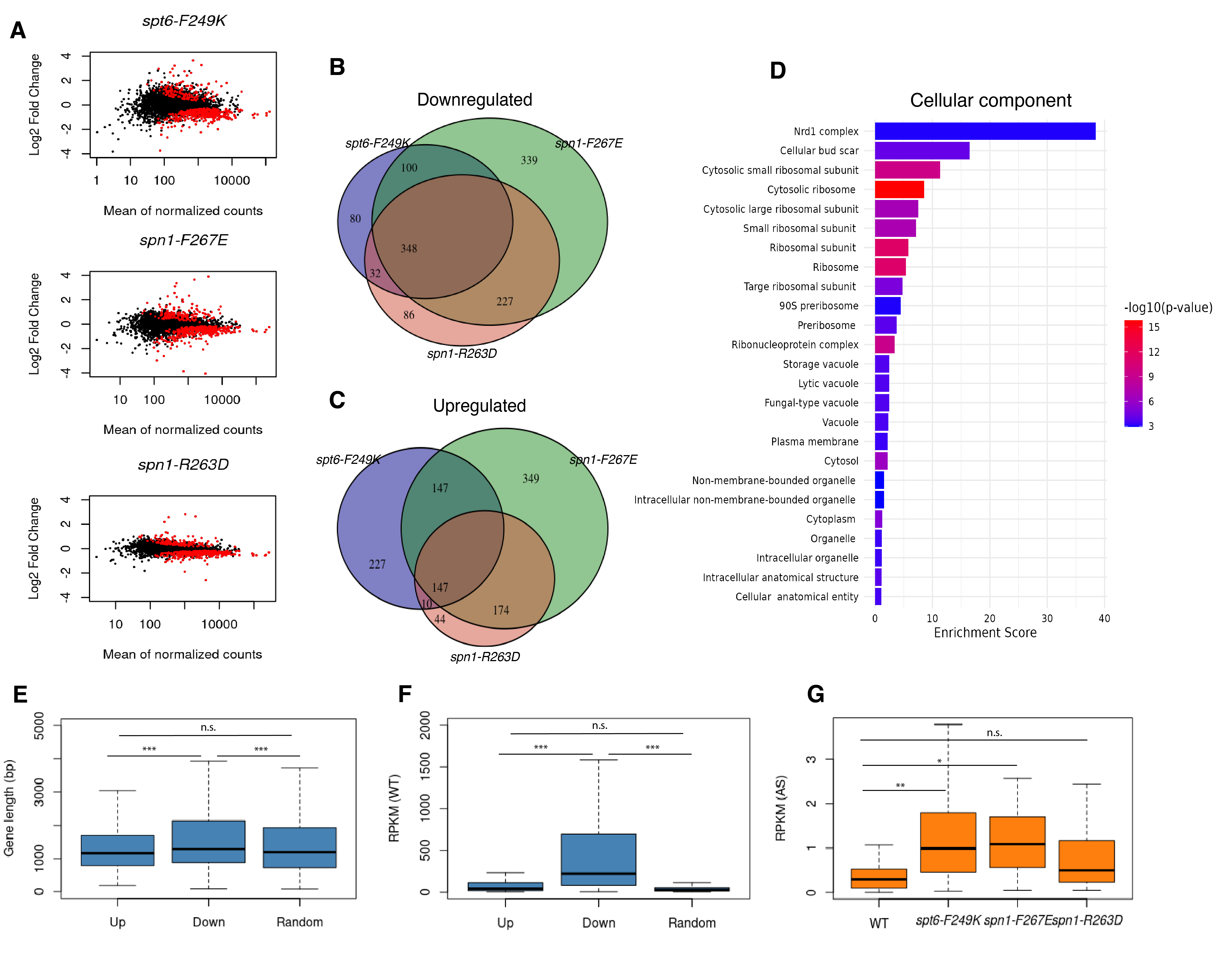
**

**Supplementary Figure 7. Disruption of Spt6-Spn1 leads to unique gene expression changes**. A) MA plots showing differential gene expression between *spt6-spn1* mutants and WT. Genes identified by DESeq to be significantly differentially expressed in all three mutants are highlighted in red. B-C**)** Venn diagrams showing the similarity of gene expression changes in the three *spt6-spn1* mutants. D) Go terms for genes that are significantly downregulated in all three *spt6-spn1* mutants. E-F) Box plot comparing the length (E) and baseline expression (F) of genes that are upregulated and downregulated across all three mutants (*** p < 0.001, Student’s t-test). G) Antisense transcription levels of 40 genes previously identified to have significantly increased antisense signal in *spt-1004* mutant (3). (** p< 0.01, * p < 0.05, Student’s t-test).

**References**

1. Szklarczyk, D., Franceschini, A., Wyder, S., Forslund, K., Heller, D., Huerta-Cepas, J., Simonovic, M., Roth, A., Santos, A., Tsafou, K. P., Kuhn, M., Bork, P., Jensen, L. J., and von Mering, C. (2015) STRING v10: protein-protein interaction networks, integrated over the tree of life. *Nucleic Acids Res* **43**, D447-452

2. Wickham, H., Navarro, D., and Lin, T. (2016) Ggplot2: Elegant graphics for data analysis *Springer International Publishing.* **2nd ed.**

3. Dronamraju, R., Kerschner, J. L., Peck, S. A., Hepperla, A. J., Adams, A. T., Hughes, K. D., Aslam, S., Yoblinski, A. R., Davis, I. J., Mosley, A. L., and Strahl, B. D. (2018) Casein Kinase II Phosphorylation of Spt6 Enforces Transcriptional Fidelity by Maintaining Spn1-Spt6 Interaction. *Cell Rep* **25**, 3476-3489 e3475
